# Mechanisms underlying *Actinobacillus pleuropneumoniae *exotoxin ApxI induced expression of IL-1β, IL-8 and TNF-α in porcine alveolar macrophages

**DOI:** 10.1186/1297-9716-42-25

**Published:** 2011-02-07

**Authors:** Zeng-Weng Chen, Maw-Sheng Chien, Nai-Yun Chang, Ter-Hsin Chen, Chi-Ming Wu, Chienjin Huang, Wei-Cheng Lee, Shih-Ling Hsuan

**Affiliations:** 1 Graduate Institute of Veterinary Pathobiology, College of Veterinary Medicine, National Chung Hsing University, 250, Kuo Kuang Road, Taichung, 402, Taiwan, ROC; 2 Graduate Institute of Microbiology and Public Health, College of Veterinary Medicine, National Chung Hsing University, 250, Kuo Kuang Road, Taichung, 402, Taiwan, ROC

## Abstract

*Actinobacillus pleuropneumoniae *(*A. pleuropneumoniae*) causes fibrino-hemorrhagic necrotizing pleuropneumonia in pigs. Production of proinflammatory mediators in the lungs is an important feature of *A. pleuropneumoniae *infection. However, bacterial components other than lipopolysaccharide involved in this process remain unidentified. The goals of this study were to determine the role of *A. pleuropneumoniae *exotoxin ApxI in cytokine induction and to delineate the underlying mechanisms. Using real-time quantitative PCR analysis, we found native ApxI stimulated porcine alveolar macrophages (PAMs) to transcribe mRNAs of IL-1β, IL-8 and TNF-α in a concentration- and time-dependent manner. Heat-inactivation or pre-incubation of ApxI with a neutralizing antiserum attenuated ApxI bioactivity to induce cytokine gene expression. The secretion of IL-1β, IL-8 and TNF-α protein from PAMs stimulated with ApxI was also confirmed by quantitative ELISA. In delineating the underlying signaling pathways contributing to cytokine expression, we observed mitogen-activated protein kinases (MAPKs) p38 and cJun NH2-terminal kinase (JNK) were activated upon ApxI stimulation. Administration of an inhibitor specific to p38 or JNK resulted in varying degrees of attenuation on ApxI-induced cytokine expression, suggesting the differential regulatory roles of p38 and JNK in IL-1β, IL-8 and TNF-α production. Further, pre-incubation of PAMs with a CD18-blocking antibody prior to ApxI stimulation significantly reduced the activation of p38 and JNK, and subsequent expression of IL-1β, IL-8 or TNF-α gene, indicating a pivotal role of β2 integrins in the ApxI-mediated effect. Collectively, this study demonstrated ApxI induces gene expression of IL-1β, IL-8 and TNF-α in PAMs that involves β2 integrins and downstream MAPKs.

## Introduction

*Actinobacillus pleuropneumoniae *(*A. pleuropneumoniae*) is the etiological agent of porcine pleuropneumonia characterized as an exudative, fibrinous, hemorrhagic, and necrotizing pneumonia along with pleuritis [1]. Multiple factors of *A. pleuropneumoniae *including lipopolysaccharide (LPS), *A. pleuropneumoniae *exotoxins (Apx), polysaccharide capsule and etc. may contribute to the disease [2-5]. Among these, Apx toxins are the major virulence factors involved in the pathogenesis of pleuropneumonia [6]. For the Apx toxins (ApxI to IV) identified so far, ApxI elicits its most significant effects on hemolysis and cytolysis [7].

Apx toxins are members of the "Repeats in Toxin" (RTX) family that are widespread in *Pasteurellaceae *which cause infectious diseases, most often in animals but also in humans [8]. RTX toxins display a cytotoxic and/or a hemolytic activity [8]. In addition, *Mannheimia haemolytica *RTX leukotoxin (Lkt) has been identified as a potent inducer on the gene expression of proinflammatory cytokines such as tumor necrosis factor alpha (TNF-α), interleukin (IL)-1, and IL-8 in bovine alveolar macrophages [9,10]. There is compelling evidence that pigs naturally or experimentally infected by *A. pleuropneumoniae *show significantly increased expression of cytokines IL-1, IL-6, IL-8 and TNF-α in lungs [4,5,11]. A study on porcine alveolar macrophages indicated multiple components of *A. pleuropneumoniae*, e.g., killed bacteria, bacterial culture supernatant, crude surface extract, or lipopolysaccharide (LPS), are potent stimulants for IL-1, IL-8 and TNF-α expression [3]. However, up to now, the role of Apx toxins in proinflammatory cytokine expression remains unidentified.

The β2 integrins have been identified to serve as a receptor for RTX toxins such as leukotoxin of *Aggregatibacter actinomycetemcomitans*, α-hemolysin of *Escherichia coli*, and Lkt of *M. haemolytica *[12-15]. β2 integrins consist of four members including leukocyte function-associated antigen 1 (LFA-1; CD11a/CD18), Mac-1 (CD11b/CD18), p150,95 (CD11c/CD18), and αdβ2 (CD11d/CD18), that play important roles in cell-matrix interaction and immune response [16]. *M. haemolytica *Lkt-induced cytotoxic effect on bovine leukocytes can be blocked by an antibody specific to ruminant CD18, suggesting a role of CD18 in Lkt-mediated cytolysis [13,15,17]. Moreover, in bovine alveolar macrophages, Lkt-induced intracellular [Ca^2+^] elevation, which is important for proinflammatory cytokine gene expression, depends on LFA-1 [9,14,18]. A recent study on ApxIII toxin revealed porcine, but not bovine or human, CD18 is necessary for mediating ApxIII-induced leukolysis [19], providing another example of a species-specific effect of RTX using the CD18 subunit of β2 integrins.

The mitogen-activated protein kinases (MAPKs) are a family of serine/threonine protein kinases that are important regulators in a variety of cellular activities [20]. A plethora of studies have revealed proinflammatory cytokines are regulated by MAPKs, such as p38, cJun NH2-terminal kinase (JNK), and extracellular signal-regulated kinase (ERK) [21-25]. Since production of proinflammatory cytokines in lungs is an important defense mechanism in response to pathogens [26], the goals of this study were to examine the effect of ApxI on cytokine expression in PAMs and to determine the potential involvement of MAPKs and β2 integrins in this event.

## Materials and methods

### Chemicals, reagents and antibodies

Brain-heart infusion (BHI) was from Becton, Dickinson and Company (Franklin Lakes, NJ, USA). The nicotinamide adenine dinucleotide (NAD), polymyxin B, 2,3-bis(2-methoxy-4-nitro-5-sulfophenyl)-2H-tetrazolium-5-carboxanilide inner salt (XTT), trypan blue, and anti-β-actin antibody were from Sigma Aldrich (Saint Louis, MO, USA). The p38 inhibitor SB203580 and JNK inhibitor SP600125 were from Calbiochem (Darmstadt, Germany). Antibody specific to active, phosphorylated p38 or JNK was from Promega (Madison, WI, USA). A blocking antibody specific to porcine CD18 was purchased from SeroTec (Kidlington, Oxfordshire, United Kingdom).

### Cell culture

Porcine alveolar macrophages (PAMs) were obtained from 3- to 6- week old healthy piglets through lavage and stored in liquid nitrogen using previously described procedures [27]. Piglets were euthanized according to the protocol approved by the Institutional Animal Care and Use Committee (IACUC) of National Chung Hsing University. For the experiments, PAMs were thawed, resuspended in culture medium RPMI-1640 supplemented with 10% fetal bovine serum (FBS), 2 mM L-glutamine, 100 U/mL penicillin, and 100 μg/mL streptomycin (Invitrogen, Carlsbad, CA, USA) and seeded into cell culture plates as indicated below.

### ApxI exotoxin preparation

*Actinobacillus pleuropneumoniae *serotype 10 (strain 13039) was a kind gift from the Animal Health Research Institute, Council of Agriculture, Republic of China. It secretes only ApxI but not ApxII and ApxIII [28]. Preparation of the ApxI was performed according to the procedures described previously [27]. Briefly, colonies of *A. pleuropneumoniae *serotype 10 on BHI agar plates containing 10 μg/mL NAD were transferred to BHI broth supplemented with 10 μg/mL NAD and cultured at 37°C for 5 h. The BHI broth was then replaced by RPMI-1640 supplemented with 2% FBS and 10 mM CaCl_2 _and cultivated for an additional 2 h. Subsequently, the bacterial culture supernatant was collected by centrifugation at 16 000 × *g *for 10 min at 4°C followed by passage through a filter with pore-size of 0.45 μm. The filtered preparation containing native exotoxin ApxI was aliquoted and stored at -70°C for further experiments. The cytotoxic activity of ApxI was determined using an XTT assay as described previously [29]. One cytotoxic unit (CU) of ApxI was defined as the quantity of toxin causing a 50% reduction in mitochondrial activity of 2 × 10^5 ^PAMs.

### Determination of LPS content in the exotoxin preparation

Limulus amebocyte lysate test (Cambrex Bio Science, Walkersville, MD, USA) was performed according to the manufacturer's instructions. It was found that the preparation containing 1 CU/mL ApxI had a level of 307 endotoxin units (EU)/mL. To minimize the effect of contaminating LPS in the exotoxin preparation, polymyxin B was added to a final concentration of 10 μg/mL throughout the study except where indicated otherwise.

### Treatment with ApxI, drugs, and antibody

PAMs were seeded to 35-mm tissue culture plates at a density of 2 × 10^6 ^cells/plate in culture medium and incubated at 37°C in 5% CO_2 _overnight. Cells were washed once and replenished with low serum medium (LSM; RPMI-1640 supplemented with 1% FBS, 2 mM L-glutamine, 100 U/mL penicillin, and 100 μg/mL streptomycin) containing 0-2 CU/mL of ApxI and incubated for 0-12 h as indicated elsewhere. To abolish the bioactivity of ApxI, exotoxin was incubated at 98°C for 1 h or pre-incubated with 20 μg/mL antiserum raised against a recombinant subunit ApxI protein for 30 min at 37°C prior to applying to PAMs in the indicated experiments [27]. In experiments examining the roles of mitogen-activated protein kinases (MAPKs) and β2 integrins on ApxI-induced cytokine expression, PAMs were incubated with LSM containing 10 μM p38 inhibitor SB203580, 10 μM JNK inhibitor SP600125, or 5-10 μg/mL anti-porcine CD18 antibody for 1 h prior to stimulation with 0.5 CU/mL of ApxI.

### Real-time quantitative PCR (RT-qPCR)

The levels of cytokine mRNA in PAMs were evaluated by RT-qPCR at 2 h post ApxI stimulation as described above. Total RNA of PAMs was extracted using High Pure RNA Isolation Kit (Roche Applied Science, Mannheim, Germany) according to the manufacturer's instructions. cDNA synthesis and RT-qPCR analysis were performed as follows.

#### cDNA synthesis

Total RNA extracted from PAMs was quantified by detection of light absorption at 260 nm using a NanoVue instrument (GE Healthcare Bio-Sciences Corp, Piscataway, NJ, USA) and ~1 μg of RNA was used for cDNA synthesis. To prepare a 20 μL reaction solution, RNA was mixed with 0.5 μg Oligo(dT)15 primer (Promega) in each PCR tube and incubated at 65°C for 10 min to ensure the denaturation of the RNA secondary structures. Subsequently, the tube was immediately placed on ice prior to adding 20 U RNasin^® ^ribonuclease inhibitor (Promega), 20 nmole dNTP, and 10 U Transcriptor Reverse Transcriptase with 1 × reaction buffer (Roche Applied Science), and a final volume adjusted to 20 μL with distilled water. cDNA synthesis was carried out at 55°C for 30 min and the activity of reverse transcriptase was inactivated by heating the solution to 85°C for 5 min. In this study, a 10-fold dilution of the synthesized cDNA was used as a template for RT-qPCR analysis.

#### Oligonucleotide primers

The sequences of oligonucleotide primers used in this study are listed in Table 1. Primers for interleukin-1 beta (IL-1β), tumor necrosis factor alpha (TNF-α), and glyceraldehyde-3-phosphate dehydrogenase (GAPDH) genes were designed based on porcine cytokine sequences obtained from the National Center for Biotechnology Information (NCBI) using Lasergene software version 5.07 (DNASTAR Inc, Madison, WI, USA). Primers for IL-8 gene were synthesized according to Cho et al. [30].

**Table 1:** Oligonucleotide primers used in this study

Genes	Sequences (5'→3')	Amplicon size (bp)	Annealing temperature (°C)	Accession No. or reference
IL-1β	Forward: GCAGTGGAGAAGCCGATGAAGA	199	58	NM_214055
	Reverse: GGCCAGCCAGCACTAGAGATTTG			
IL-8	Forward: TTTCTGCAGCTCTCTGTGAGG	269	48	[30]
	Reverse: CTGCTGTTGTTGTTGCTTCTC			
TNF-α	Forward: CGCATCGCCGTCTCCTACCA	202	58	NM_214022
	Reverse: GCCCAGATTCAGCAAAGTCCAGAT			
GAPDH	Forward: GGCTGCCCAGAACATCATCC	195	58	AF309651
	Reverse: GACGCCTGCTTCACCACCTTCTTG			

#### Real-time quantitative PCR amplification

Real-time PCR amplification was performed using the LightCycler^® ^480 SYBR green I master with a LightCycler^® ^480 instrument (Roche Applied Science). Briefly, to prepare the 2 × master mix, primers of the target gene were added into the LightCycler^® ^480 SYBR green I master to a concentration of 0.4 μM. Five microliters of 2 × master mix was further aliquoted to every well of the 96-well reaction plate, followed by addition of 5 μL of the cDNA sample. The reactions were carried out as an initial pre-incubation at 95°C for 5 min, followed by 45 amplification cycles of: 95°C for 10 s, 58°C or 48°C for 10 s, and 72°C for 15 s. Melting curve analysis was performed immediately after amplification from 65 to 95°C with continuous fluorescence acquisition. In each reaction, the cycle number at which the fluorescence rises appreciably above the background fluorescence is determined as crossing point (CP). In this study, the background fluorescence and the CP values are automatically calculated by the software (version 1.5).

#### Calculation of relative gene expression

The level of cytokine gene expression was analyzed using the "Delta-delta method" for relative quantification [31]. The expression of the selected cytokine gene was normalized to that of the reference GAPDH gene using the equation 2^-[ΔCP sample - ΔCP control] ^= 2 ^-ΔΔCP ^and further converted to relative mRNA expression. In experiments assessing the kinetics of ApxI on cytokine gene expression, the relative mRNA expression was further normalized to the percent of cell survival at each time point.

### Determination of the protein levels of cytokines

To quantify the protein levels of cytokines, 2 × 10^6 ^PAMs were stimulated with 1 CU/mL of ApxI for 4 h. In the inhibitor experiments, 1 × 10^6 ^PAMs were incubated with 0.5 CU/mL ApxI for 8 h. After treatments, culture supernatants were collected following centrifugation at 700 × *g *for 10 min. The levels of cytokines IL-1β, IL-6, IL-8, and TNF-α in the culture supernatants were determined by quantitative DuoSet^® ^ELISA kits (R&D Systems, Minneapolis, MN, USA) according to the manufacturer's instructions.

### Trypan blue exclusion test

The survival rate of PAMs was assessed using a trypan blue exclusion test [32]. Briefly, after ApxI treatment, PAMs were collected by trypsinization, stained with 0.1% trypan blue, and observed using a microscope. Cells stained blue were scored as non-viable. At least 500 cells were counted for each treatment of at least triplicate determinations. Percent of cell survival was calculated as follow: 100 × [1 - (% cell death at each time point - % cell death at 0 h)].

### Western blot analysis

In experiments examining MAPK activation, 2 × 10^6 ^cells PAMs in 35-mm tissue culture plates were stimulated with 0.5 CU/mL ApxI for 1 h. Thereafter, cells were washed once with ice-cold phosphate buffered saline (PBS) and lysed in a lysis buffer (1% Triton X-100, 20 mM Tris-Cl, 137 mM NaCl, 25 mM β-glycerophosphate, 2 mM NaPPi, 1 mM Na_3_VO_4_, 10% glycerol, 2 mM EDTA, 10 μM leupeptin, 0.77 μM aprotinin, 0.5 mM DTT, 10 mM PMSF, and 2 mM benzamidine; pH 7.4) [33]. Cell lysates were centrifuged at 15 000 × *g *for 10 min and supernatants harvested. The protein concentration of the cell lysate was determined using the Bradford assay (Bio-Rad Laboratories, Hercules, CA, USA). Twenty to thirty μg of each lysate was analyzed by Western blot analysis using an antibody specific to active, phosphorylated p38 or JNK. Immunoblots were reprobed with an anti-β-actin antibody as a loading control. The intensity of the active p38 or JNK was quantified using ImageJ software, version 1.37v (National Institutes of Health), and normalized to the intensity of the loading control β-actin.

### Statistical analysis

Data were obtained from three independent experiments of at least triplicate determinations. Statistical analysis was performed using one-way analysis of variance (ANOVA). The error bars represent the standard error of mean (SEM).

## Results

### *Actinobacillus pleuropneumoniae *ApxI induces expression of proinflammatory cytokine genes in porcine alveolar macrophages

**Figure 1:**
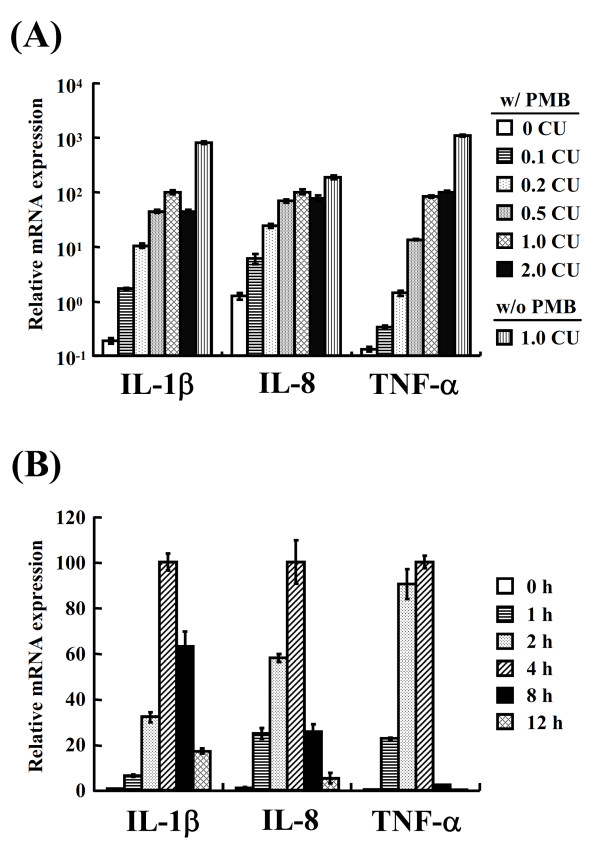
***A. pleuropneumoniae *exotoxin ApxI induces IL-1β, IL-8 and TNF-α mRNA expression of porcine alveolar macrophages (PAMs) in a concentration- and time-dependent manner**. (A) PAMs were stimulated with 0-2 CU/mL ApxI in the presence (w/) or absence (w/o) of polymyxin B (PMB) for 2 h. Total RNA of PAMs was extracted for cDNA synthesis and subjected to RT-qPCR. (B) PAMs were treated with 1 CU/mL ApxI in the presence of PMB for 0-12 h and subjected to RT-qPCR. The expression levels of cytokines were further normalized to corresponding survival rate of PAMs at each time point. Data are representative of three independent experiments of at least triplicate determinations. The error bars are SEM.

To evaluate the effect of *A. pleuropneumoniae *serotype 10-derived ApxI on proinflammatory cytokine gene expression, porcine alveolar macrophages (PAMs) were incubated with 0-2 CU/mL of ApxI for 2 h and subjected to real-time quantitative PCR (RT-qPCR) analysis. Significant elevation of mRNA levels of IL-1β, IL-8 and TNF-α was noted in PAMs treated with 0.2 to 2 CU/mL of ApxI (*p *< 0.001) (Figure 1A). The levels of cytokine mRNA increased as the concentration of ApxI elevated to 1 CU/mL, indicating a concentration-dependent effect of ApxI. Substantial levels of mRNA expression were noted in cells treated with ≥ 0.5 CU/mL (Figure 1A). In subsequent experiments, ApxI at 0.5 or 1 CU/mL was used throughout.

In addition, cytokine expression was compared in the absence and presence of an LPS-blocker polymyxin B (PMB). It was noted that 1 CU/mL ApxI preparation without PMB elicited mRNA expression of IL-1β, IL-8, and TNF-α at a level ~8-, 2-, and 13-fold higher than that treated with ApxI in the presence of PMB, respectively (Figure 1A).

To assess the kinetics of ApxI on proinflammatory cytokine gene expression, PAMs were treated with 1 CU/mL of ApxI for varied time periods and mRNA levels were evaluated using RT-qPCR. The mRNA levels of IL-1β, IL-8 and TNF-α elevated at 1 h following ApxI treatment (Figure 1B). The maximal levels of mRNA expression of all three cytokines were observed at 4 h. At 8 or 12 h, the mRNA levels of all these cytokines were less than 63% of the maximal levels (Figure 1B). Collectively, these findings demonstrate *A. pleuropneumoniae *serotype 10-derived ApxI induces IL-1β, IL-8 and TNF-α gene expression in PAMs in a concentration- and time-dependent manner.

### Exotoxin-induced proinflammatory cytokine gene expression is attributed to ApxI bioactivity

**Figure 2:**
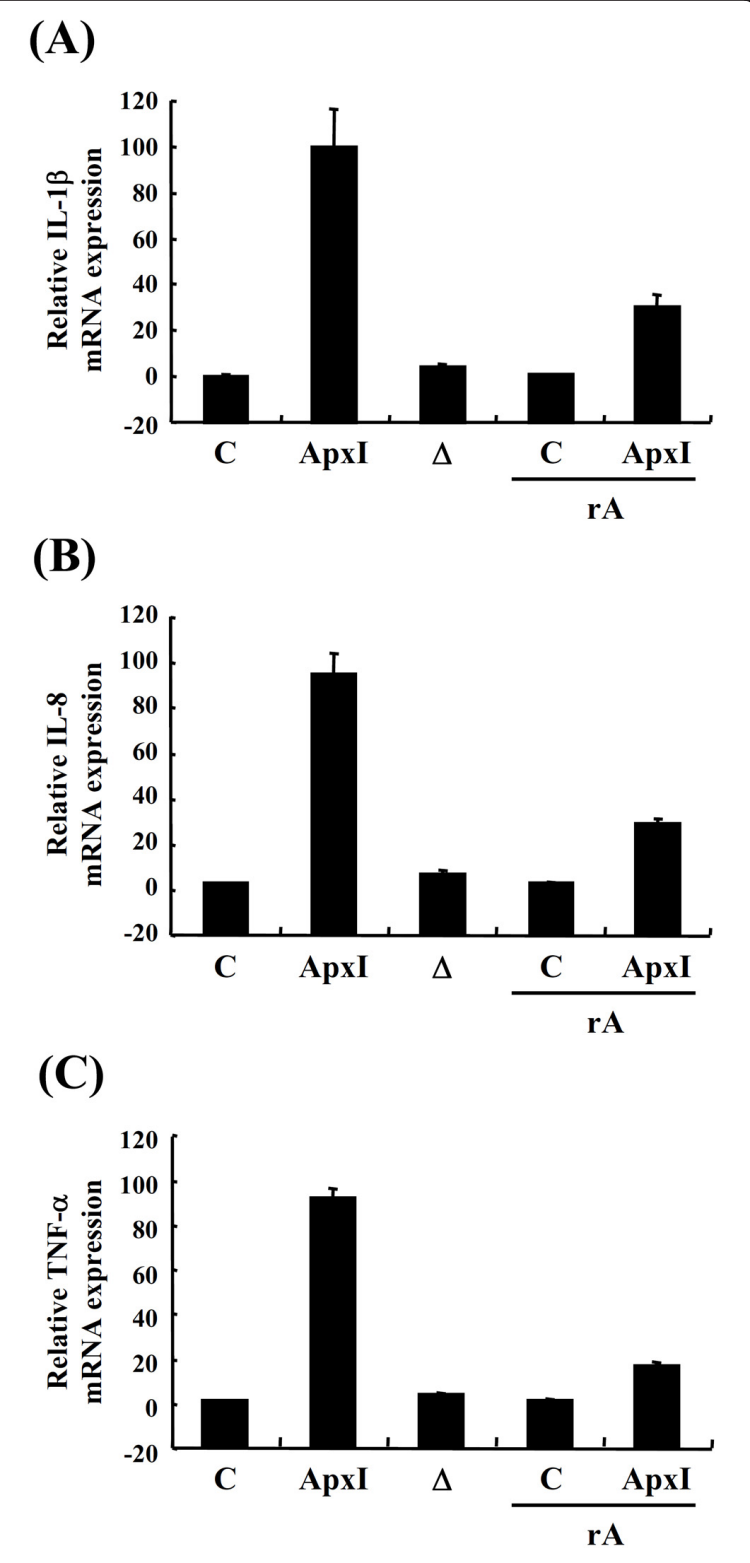
**Bioactivity of ApxI is required for induction of proinflammatory cytokine gene expression**. PAMs were treated with 0.5 CU/mL of ApxI, heat-inactivated ApxI (Δ), or ApxI pre-incubated with an antiserum raised against recombinant subunit ApxI protein (rA). Medium without ApxI toxin served as a negative control (C). Two hours after stimulation, the total RNA of PAMs was extracted for RT-qPCR analysis. Data are representative of three independent experiments of at least triplicate determinations.

To demonstrate cytokine gene expression in PAMs is truly attributable to the bioactivity of ApxI, ApxI was heat-inactivated (ΔApxI) or pre-incubated with an antiserum raised against a recombinant subunit ApxI protein [27] before application to PAMs and subjected to RT-qPCR analysis. PAMs treated with ΔApxI had mRNA level of IL-1β, IL-8 or TNF-α that was not significantly different from cells without treatment (Figure 2). Moreover, PAMs treated with serum-neutralized ApxI showed a 70-84% attenuation in IL-1β, IL-8 or TNF-α mRNA expression level, indicating IL-1β, IL-8 or TNF-α gene expression is ascribed to the bioactivity of ApxI.

**Figure 3:**
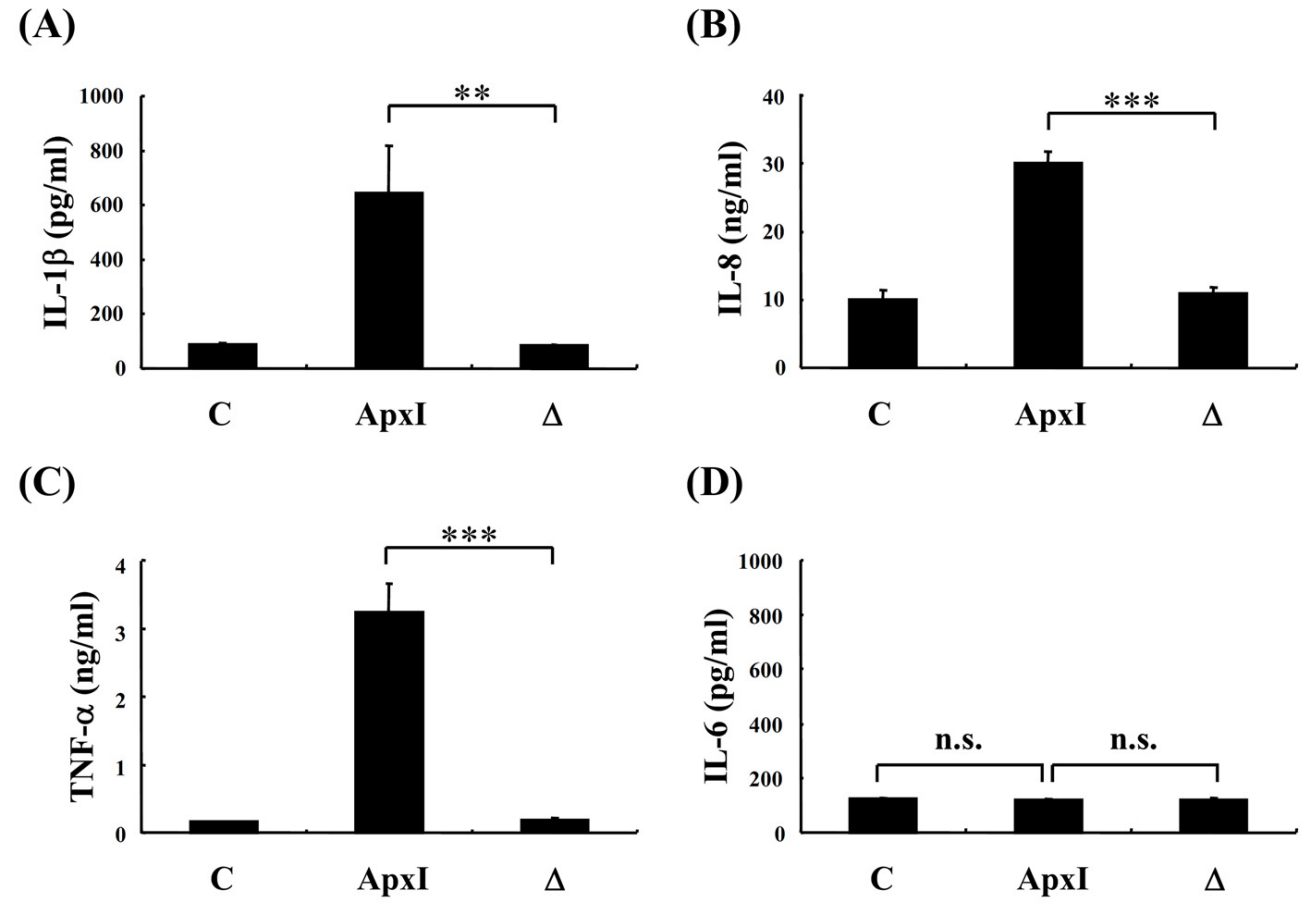
**Protein levels of proinflammatory cytokines secreted from PAMs treated with ApxI**. PAMs were stimulated with 1 CU/mL of ApxI, heat-inactivated ApxI (Δ), or control medium (C) for 4 h. Subsequently, culture supernatants were collected and the protein levels of IL-1β (A), IL-8 (B), TNF-α (C) and IL-6 (D) determined by ELISA. The results are the average of three independent experiments of triplicate determinations. **p < 0.01; ***p < 0.001; n.s.: no significant difference from each other.

To confirm the effects of ApxI on the protein level of cytokine induction, PAMs were incubated with 1 CU/mL ApxI for 4 h and cell culture supernatants analyzed using quantitative ELISA. Incubation of ApxI resulted in secretion of ~650 pg/mL of IL-1β, 30 ng/mL of IL-8, and 3.2 ng/mL of TNF-α from PAMs (Figure 3). Cells stimulated with ΔApxI had a level of 84 pg/mL of IL-1β in culture supernatant that was not significantly different from cells without treatment (Figure 3A). A similar pattern was also observed for the protein levels of IL-8 and TNF-α in cells treated with ΔApxI and non-treated cells (Figures 3B and 3C). Interestingly, only the basal level (~120 pg/mL) of IL-6 was detected in ApxI-treated PAMs, which was similar to that in cells without treatment or treated with ΔApxI (Figure 3D).

### Involvement of mitogen-activated protein kinases (MAPKs) in ApxI-induced proinflammatory cytokine gene expression

**Figure 4:**
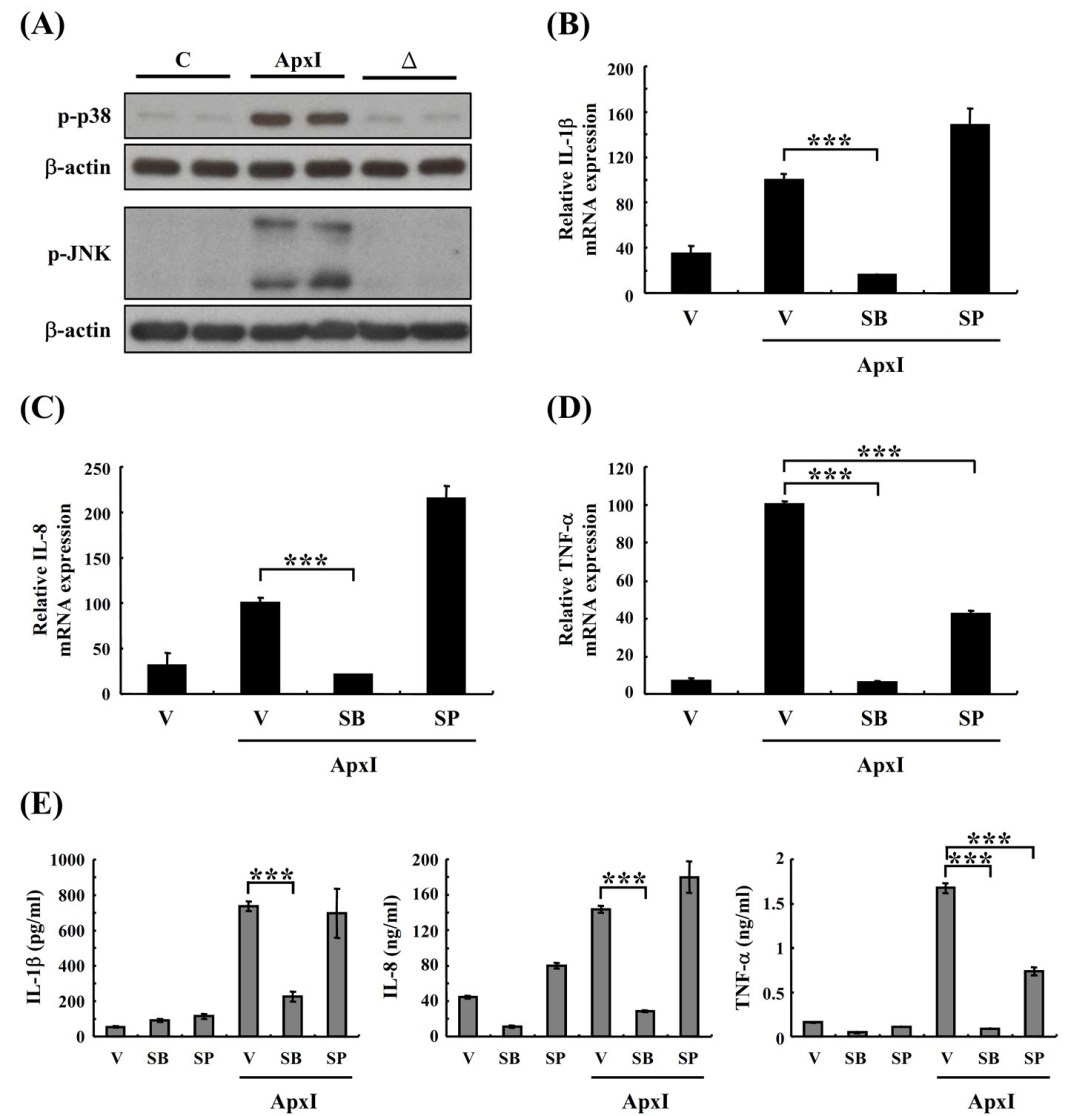
**p38 and JNK mediate ApxI-induced proinflammatory cytokine production**. (A) PAMs were stimulated with 0.5 CU/mL of ApxI, heat-inactivated ApxI (Δ), or control medium (C) for 1 h. Subsequently, cells were lysed and subjected to Western blot analysis using an antibody specific to phospho-p38 (p-p38) or phospho-JNK (p-JNK). (B-D) PAMs were pre-incubated with a p38 inhibitor SB203580 (SB), a JNK inhibitor SP600125 (SP), or vehicle DMSO (V) for 1 h, followed by stimulation with 0.5 CU/mL ApxI for 2 h. PAMs were subjected to RT-qPCR analysis for mRNA levels of IL-1β (B), IL-8 (C), and TNF-α (D). (E) PAMs were pre-incubated with SB, SP, or DMSO for 1 h, followed by stimulation with 0.5 CU/mL of ApxI for 8 h. Subsequently, the culture supernatants were collected and the protein levels of IL-1β, IL-8, and TNF-α were determined by ELISA. Data are representative of three independent experiments of at least triplicate determinations. ***p < 0.001.

A plethora of studies indicate members of MAPK family play important roles in proinflammatory cytokine induction [21-25]. These findings prompted us to examine the roles of MAPK members p38 and JNK in ApxI effect. PAMs stimulated with 0.5 CU/mL of ApxI for 1 h were subjected to Western blot analysis for active p38 and JNK. ApxI treatment induced an appreciable increase in both active, phosphorylated p38 and JNK in PAMs (Figure 4A). Low levels of phosphorylated p38 were noted in cells treated with ΔApxI that did not significantly differ from non-treated control cells, and there was no detectable phosphorylated JNK in ΔApxI-treated or control cells, suggesting the activation of JNK and p38 MAPK is attributable to ApxI.

To further assess the role of p38 or JNK in ApxI-induced cytokine expression, PAMs were pre-treated with an inhibitor specific to p38 (SB20358) or JNK (SP600125), or vehicle DMSO for 1 h, followed by stimulation with 0.5 CU/mL ApxI for 2 h, and subjected to RT-qPCR analysis. Treatment of p38 inhibitor prior to ApxI stimulation significantly attenuated IL-1β mRNA level by 83%. However, the JNK inhibitor did not attenuate the mRNA expression of IL-1β (Figure 4B). Similarly, ApxI-induced IL-8 mRNA expression was inhibited by p38 inhibitor up to ~85%, but not by JNK inhibitor (Figure 4C). Distinguishably, both p38 and JNK inhibitors blocked ApxI-induced TNF-α mRNA expression by 92% and 54%, respectively (Figure 4D). To confirm our findings, the cytokines secreted from PAMs pre-treated with inhibitors and stimulated with ApxI were also evaluated by quantitative ELISA. In the presence of the p38 inhibitor, the protein levels of IL-1β, IL-8 and TNF-α were significantly inhibited by 69-95% compared to cells without inhibitor pre-treatment and stimulated with ApxI (Figure 4E). Notably, the JNK inhibitor attenuated the protein level of TNF-α, but not IL-1β or IL-8 (Figure 4E). These data suggest that MAPK p38 plays a major role in ApxI-induced IL-1β, TNF-α, and IL-8 expression, while JNK participates only in ApxI-induced TNF-α expression.

### β2 integrins mediate MAPK activation and subsequent proinflammatory cytokine gene expression

**Figure 5:**
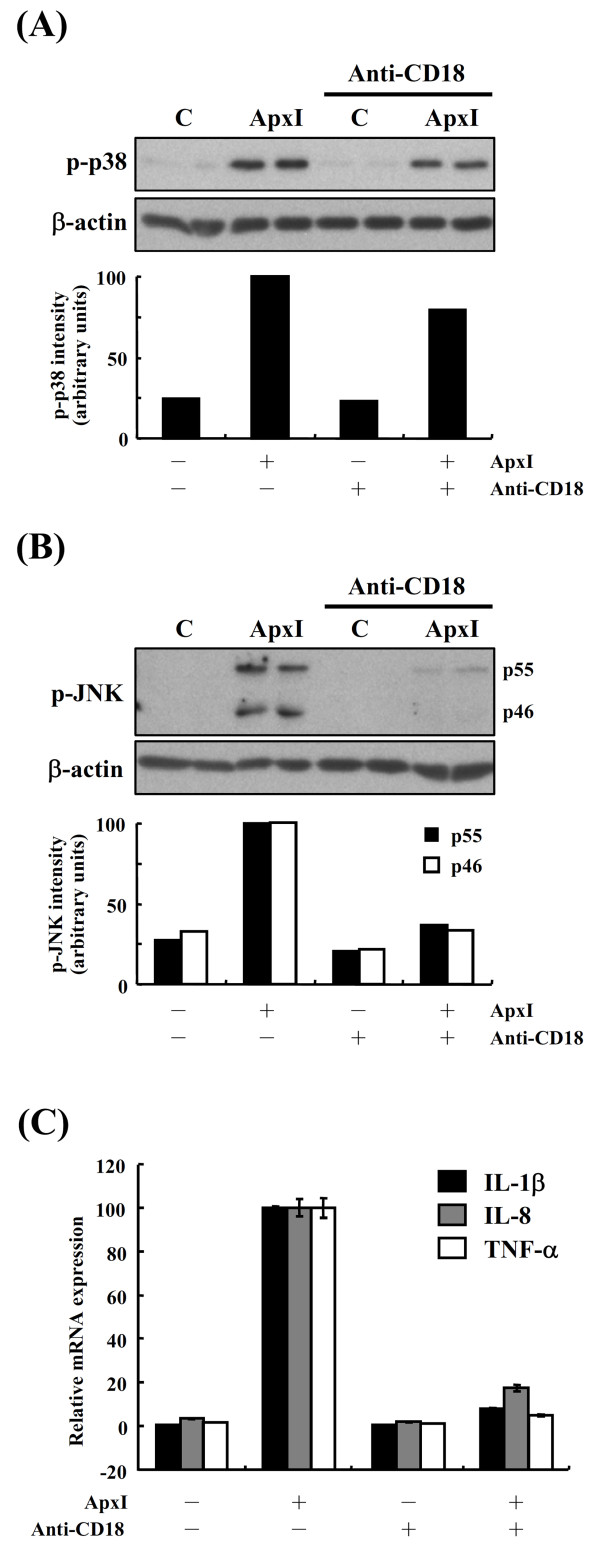
**CD18 mediates p38 and JNK activation and proinflammatory cytokine mRNA expression**. (A and B) PAMs were pre-incubated with CD18 blocking antibody (Anti-CD18) for 1 h prior to stimulation with 0.5 CU/mL ApxI or control medium (C) for an additional 1 h. Thereafter, cells were lysed and subjected to Western blot analysis with an antibody to phospho-p38 (p-p38) or phospho-JNK (p-JNK). The average intensity of p-p38 or p-JNK isoforms (p46 and p55) was quantified and normalized to the intensity of β-actin (A and B, lower panel). (C) PAMs were pre-incubated with anti-CD18 antibody and stimulated with 0.5 CU/mL ApxI for 2 h, followed by RT-qPCR analysis of cytokine mRNA expression. Data are representative of three independent experiments of at least triplicate determinations.

The common subunit CD18 of β2 integrins is necessary for *A. pleuropneumoniae *ApxIII-induced leukolysis [19]. To examine whether ApxI-induced MAPK activation was attributable to ApxI-β2 integrin interaction, a blocking antibody of porcine CD18 was applied to PAMs 1 h prior to stimulation with 0.5 CU/mL ApxI and analyzed by Western blot analysis. In the presence of CD18 antibody, ApxI-induced p38 activation was significantly inhibited by 20-30% (Figure 5A). Cells treated with the control medium or medium containing CD18 antibody displayed only a basal level of phosphorylated p38. Similarly, blocking of CD18 on PAMs resulted in attenuation of ~65% ApxI-activated JNK phosphorylation (Figure 5B). However, phosphorylated JNK was not observed in cells treated with the control medium or medium containing CD18 antibody. Further, to evaluate the effect of ApxI-β2 integrin interaction on cytokine mRNA expression, PAMs were pre-incubated with CD18 antibody, stimulated with 0.5 CU/mL ApxI for 2 h, and subjected to RT-qPCR analysis. Blocking of CD18 molecule attenuated IL-1β, IL-8 and TNF-α mRNA levels by 82-95%. Cells treated with control medium or medium containing CD18 antibody displayed similar basal levels of IL-1β, IL-8 and TNF-α mRNA (Figure 5C). Collectively, these results indicate CD18 plays a pivotal role in ApxI-mediated MAPK activation and expression of proinflammatory cytokine genes in PAMs.

## Discussion

Inflammation plays an essential role in respiratory defense mechanism [26]. In porcine pleuropneumonia, multiple components and virulence factors of *A. pleuropneumoniae *are suggested to contribute to the production of proinflammatory cytokines [3]. Baarsch et al. demonstrated alveolar lavage cells from pigs endotracheally inoculated with a low-virulence strain of *A. pleuropneumoniae *serotype 1, i.e., lacking cytotoxic and hemolytic activities, showed similar mRNA level of TNF, IL-1 or IL-8 compared to buffer treated pigs [5]. Moreover, heat-treatment of crude *A. pleuropneumoniae *bacterial culture supernatant attenuated its ability to induce TNF-α and IL-1 expression in porcine alveolar macrophages (PAMs), suggesting heat-labile components were involved in such stimulation [3]. In this study, we present direct evidence demonstrating a single species of exotoxin ApxI, derived from *A. pleuropneumoniae *serotype 10, induces the expression and production of proinflammatory cytokines IL-1β, IL-8 and TNF-α in PAMs.

Also, we found that, at low concentration such as 0.5 or 1 CU/mL, ApxI did not cause significant cell death within 4 h, and > 68% of PAMs survived at 12 h of stimulation (data not shown). Since alveolar macrophages are one of the first line defense cells in lungs, at the early stage of *A. pleuropneumoniae *infection, PAMs may be a source of inflammatory mediators contributing to innate immunity. Nevertheless, PAMs may not be the only source of proinflammatory cytokines during *A. pleuropneumoniae *infection. For instance, Baarsch et al. demonstrated expression of cytokine IL-6 in the lung tissue of pigs inoculated with live *A. pleuropneumoniae *bacteria [5]. However, fibroblasts and epithelial cells, but not alveolar macrophages, might be the source of IL-6 [5]. This is consistent with our findings showing PAMs did not express IL-6 upon ApxI stimulation. Therefore, whether ApxI stimulates inflammatory cytokine IL-1β, IL-8 or TNF-α in other cell types remains to be clarified.

Overproduction of proinflammatory mediators in lungs can harm animals [26]. Based on our findings in this study, it is tempting to hypothesize, in addition to the hemolytic and cytolytic effects of ApxI, the toxin may exacerbate lung damages caused by *A. pleuropneumoniae *through the induction of proinflammatory cytokines. However, to avoid overemphasizing the effect of ApxI, it should be noted that the cell wall component of Gram negative bacteria, i.e., LPS, induces plethora of inflammatory and immunological effects in various cell types [34], such as TNF, IL-1 and IL-8 production by porcine macrophages [35-37]. Moreover, Ramjeet et al. had demonstrated that the cytotoxicity of ApxI and ApxII was enhanced through interaction with LPS [38]. In our study, we found that in the absence of PMB, ApxI preparation induced significantly higher levels of proinflammatory cytokine expression as compared to treatment of ApxI in the presence of PMB (Figure 1A). These findings indicate both LPS and ApxI are cytokine inducing factors. However, whether LPS contributes to a synergistic or additive effect on ApxI-induced cytokine expression requires further investigation.

Mitogen-activated protein kinases (MAPKs) are important signaling molecules involved in the expression of inflammatory mediators in various types of cells [21-25]. Upon MAPK activation, down-stream targets including transcription factors are phosphorylated and activated that in turn bind to the promoter region of target genes [20]. Cooperative involvement of multiple transcription factors is a common theme in the gene regulation of single cytokine. It has been demonstrated activating protein-1 (AP-1; consisting of heterodimers of c-Jun, activating transcription factor-2 (ATF-2), c-Fos, and Jun dimerization protein (JDP)), nuclear factor kappa B (NF-κB), and CAAT/enhancer-binding protein (C/EBP) participate in transcriptional control of IL-8 gene [21]. Similarly, the expression of TNF-α gene is also regulated by C/EBP, NF-κB, and AP-1 (c-Jun/ATF-2) [39]. In addition, it is well-established MAPKs may have overlapping substrate specificity on transcription factors [20]. For example, c-Jun is phosphorylated and activated specifically by JNK, while ATF-2 can be phosphorylated by both JNK and p38 MAPKs [33,40,41]. Nevertheless, the involvement of MAPKs in RTX-induced cytokine gene expression has not been identified.

In the present study, we showed both p38 and JNK were substantially activated after ApxI stimulation, and these two signaling molecules played differential regulatory roles in cytokine gene expression. Our observations that p38 inhibitor (SB203580) attenuated the expression of IL-1β, IL-8 and TNF-α, while JNK inhibitor (SP600125) attenuated the expression only on TNF-α gene, suggest p38 plays a more important role in ApxI-induced IL-1β, IL-8 and TNF-α expression. These findings are similar to previous studies showing SB203580 inhibited gene expression of IL-1β, IL-8 and TNF-α [21,23,25], while SP600125 inhibited gene expression of TNF-α, but not IL-1β or IL-8 [42]. However, the question of which transcription factors are involved in ApxI-induced cytokine expression and the regulatory role of MAPKs on these factors remain to be determined.

CD18 of β2 integrins has been proposed as a receptor for ApxIII-mediated leukolysis [19]. In this study, the observations that blocking of CD18 on PAMs resulted in attenuation of ApxI-induced p38 and JNK activation and subsequent cytokine gene expression suggest ApxI-β2 integrin interaction contributes to these events. Nevertheless, current knowledge of signaling pathways underlying β2 integrin-induced MAPK activation is very limited. Studies by Hsuan et al. indicated *M. haemolytica *Lkt-induced intracellular [Ca^2+^] ([Ca^2+^]_i_) elevation in bovine alveolar macrophages is mediated through a G-protein-coupled mechanism [18], and [Ca^2+^]_i _elevation is crucial for proinflammatory cytokine gene expression [9]. Consequently, a study by Jeyaseelan et al. demonstrated Lkt-induced [Ca^2+^]_i _elevation depends on LFA-1 [14]. It has been reported MAPKs are activated by a small G-protein Ras downstream G-protein-coupled receptor (GPCR) signaling pathway [20,43]. The question of whether ApxI-induced MAPK activation also occurs through a calcium-dependent, G-protein-coupled mechanism leading to cytokine gene expression requires further investigation.

In summary, this study provides evidence demonstrating ApxI is able to induce the expression of proinflammatory cytokines IL-1β, IL-8 and TNF-α in porcine alveolar macrophages. This is the first report to demonstrate p38 and JNK play differential regulatory roles in ApxI-induced cytokine gene expression through a CD18-dependent pathway.

## Competing interests

The authors declare that they have no competing interests.

## Authors' contributions

ZWC performed the experiments and wrote the paper. MSC and SLH designed the experiments. NYC, THC, and CMW assisted with the experiments. CH and WCL assisted with data analysis. SLH developed the original concepts and wrote the paper. All authors read and approved the final manuscript.

## References

[B1] Chiers K De Waele T Pasmans F Ducatelle R Haesebrouck F Virulence factors of *Actinobacillus pleuropneumoniae *involved in colonization, persistence and induction of lesions in its porcine host Vet Res 2010 41 65 10.1051/vetres/2010037 20546697 PMC2899255

[B2] Inzana TJ Virulence properties of *Actinobacillus pleuropneumoniae* Microb Pathog 1991 11 305 316 10.1016/0882-4010(91)90016-4 1816486

[B3] Huang H Potter AA Campos M Leighton FA Willson PJ Haines DM Yates WD Pathogenesis of porcine *Actinobacillus pleuropneumoniae*, part II: roles of proinflammatory cytokines Can J Vet Res 1999 63 69 78 9918337 PMC1189518

[B4] Choi C Kwon D Min K Chae C *In-situ *hybridization for the detection of inflammatory cytokines (IL-1, TNF-α and IL-6) in pigs naturally infected with *Actinobacillus pleuropneumoniae* J Comp Pathol 1999 121 349 356 10.1053/jcpa.1999.0332 10542124

[B5] Baarsch MJ Scamurra RW Burger K Foss DL Maheswaran SK Murtaugh MP Inflammatory cytokine expression in swine experimentally infected with *Actinobacillus pleuropneumoniae* Infect Immun 1995 63 3587 3594 7642295 10.1128/iai.63.9.3587-3594.1995 PMC173498

[B6] Bosse JT Janson H Sheehan BJ Beddek AJ Rycroft AN Kroll JS Langford PR *Actinobacillus pleuropneumoniae*: pathobiology and pathogenesis of infection Microbes Infect 2002 4 225 235 10.1016/S1286-4579(01)01534-9 11880056

[B7] Kamp EM Popma JK Anakotta J Smits MA Identification of hemolytic and cytotoxic proteins of *Actinobacillus pleuropneumoniae *by use of monoclonal antibodies Infect Immun 1991 59 3079 3085 1879932 10.1128/iai.59.9.3079-3085.1991 PMC258137

[B8] Frey J Kuhnert P RTX toxins in *Pasteurellaceae* Int J Med Microbiol 2002 292 149 158 10.1078/1438-4221-00200 12398206

[B9] Hsuan SL Kannan MS Jeyaseelan S Prakash YS Malazdrewich C Abrahamsen MS Sieck GC Maheswaran SK *Pasteurella haemolytica *leukotoxin and endotoxin induced cytokine gene expression in bovine alveolar macrophages requires NF-κB activation and calcium elevation Microb Pathog 1999 26 263 273 10.1006/mpat.1998.0271 10222211

[B10] Yoo HS Rajagopal BS Maheswaran SK Ames TR Purified *Pasteurella haemolytica *leukotoxin induces expression of inflammatory cytokines from bovine alveolar macrophages Microb Pathog 1995 18 237 252 10.1016/S0882-4010(05)80001-4 7476090

[B11] Baarsch MJ Foss DL Murtaugh MP Pathophysiologic correlates of acute porcine pleuropneumonia Am J Vet Res 2000 61 684 690 10.2460/ajvr.2000.61.684 10850846

[B12] Lally ET Kieba IR Sato A Green CL Rosenbloom J Korostoff J Wang JF Shenker BJ Ortlepp S Robinson MK Billings PC RTX toxins recognize a β2 integrin on the surface of human target cells J Biol Chem 1997 272 30463 30469 10.1074/jbc.272.48.30463 9374538

[B13] Ambagala TC Ambagala AP Srikumaran S The leukotoxin of *Pasteurella haemolytica *binds to β2 integrins on bovine leukocytes FEMS Microbiol Lett 1999 179 161 167 10481101 10.1111/j.1574-6968.1999.tb08722.x

[B14] Jeyaseelan S Hsuan SL Kannan MS Walcheck B Wang JF Kehrli ME Lally ET Sieck GC Maheswaran SK Lymphocyte function-associated antigen 1 is a receptor for *Pasteurella haemolytica *leukotoxin in bovine leukocytes Infect Immun 2000 68 72 79 10.1128/IAI.68.1.72-79.2000 10603370 PMC97103

[B15] Li J Clinkenbeard KD Ritchey JW Bovine CD18 identified as a species specific receptor for *Pasteurella haemolytica *leukotoxin Vet Microbiol 1999 67 91 97 10.1016/S0378-1135(99)00040-1 10414364

[B16] Evans R Patzak I Svensson L De Filippo K Jones K McDowall A Hogg N Integrins in immunity J Cell Sci 2009 122 215 225 10.1242/jcs.019117 19118214

[B17] Deshpande MS Ambagala TC Ambagala AP Kehrli ME Jr Srikumaran S Bovine CD18 is necessary and sufficient to mediate *Mannheimia *(*Pasteurella*) *haemolytica *leukotoxin-induced cytolysis Infect Immun 2002 70 5058 5064 10.1128/IAI.70.9.5058-5068.2002 12183553 PMC128227

[B18] Hsuan SL Kannan MS Jeyaseelan S Prakash YS Sieck GC Maheswaran SK *Pasteurella haemolytica *A1-derived leukotoxin and endotoxin induce intracellular calcium elevation in bovine alveolar macrophages by different signaling pathways Infect Immun 1998 66 2836 2844 9596757 10.1128/iai.66.6.2836-2844.1998 PMC108279

[B19] Vanden Bergh PG Zecchinon LL Fett T Desmecht D Porcine CD18 mediates *Actinobacillus pleuropneumoniae *ApxIII species-specific toxicity Vet Res 2009 40 33 10.1051/vetres/2009016 19356397 PMC2701182

[B20] Kyriakis JM Avruch J Mammalian mitogen-activated protein kinase signal transduction pathways activated by stress and inflammation Physiol Rev 2001 81 807 869 11274345 10.1152/physrev.2001.81.2.807

[B21] Hoffmann E Dittrich-Breiholz O Holtmann H Kracht M Multiple control of interleukin-8 gene expression J Leukoc Biol 2002 72 847 855 12429706

[B22] Kaminska B MAPK signalling pathways as molecular targets for anti-inflammatory therapy--from molecular mechanisms to therapeutic benefits Biochim Biophys Acta 2005 1754 253 262 16198162 10.1016/j.bbapap.2005.08.017

[B23] Baldassare JJ Bi Y Bellone CJ The role of p38 mitogen-activated protein kinase in IL-1β transcription J Immunol 1999 162 5367 5373 10228013

[B24] Han J Lee JD Bibbs L Ulevitch RJ A MAP kinase targeted by endotoxin and hyperosmolarity in mammalian cells Science 1994 265 808 811 10.1126/science.7914033 7914033

[B25] Lee JC Laydon JT McDonnell PC Gallagher TF Kumar S Green D McNulty D Blumenthal MJ Heys JR Landvatter SW Strickler JE McLaughlin MM Siemens IR Fisher SM Livi GP White JR Adams JL Young PR A protein kinase involved in the regulation of inflammatory cytokine biosynthesis Nature 1994 372 739 746 10.1038/372739a0 7997261

[B26] Thacker EL Lung inflammatory responses Vet Res 2006 37 469 486 10.1051/vetres:2006011 16611559

[B27] Chien MS Chan YY Chen ZW Wu CM Liao JW Chen TH Lee WC Yeh KS Hsuan SL *Actinobacillus pleuropneumoniae *serotype 10 derived ApxI induces apoptosis in porcine alveolar macrophages Vet Microbiol 2009 135 327 333 10.1016/j.vetmic.2008.09.071 19013727

[B28] Frey J Virulence in *Actinobacillus pleuropneumoniae *and RTX toxins Trends Microbiol 1995 3 257 261 10.1016/S0966-842X(00)88939-8 7551637

[B29] Chung WB Backstrom LR McDonald J Collins MT The (3-(4,5-dimethylthiazol-2-yl)-2,5-diphenyltetrazolium) colorimetric assay for the quantitation of *Actinobacillus pleuropneumoniae *cytotoxin Can J Vet Res 1993 57 159 165 8358675 PMC1263617

[B30] Cho WS Chae C Expression of inflammatory cytokines (TNF-α, IL-1, IL-6 and IL-8) in colon of pigs naturally infected with *Salmonella typhimurium *and *Salmonella choleraesuis* J Vet Med A Physiol Pathol Clin Med 2003 50 484 487 15157014 10.1111/j.1439-0442.2004.00588.x

[B31] Livak KJ Schmittgen TD Analysis of relative gene expression data using real-time quantitative PCR and the 2^-ΔΔ^^*C*^^T ^method Methods 2001 25 402 408 10.1006/meth.2001.1262 11846609

[B32] Rycroft AN Williams D Cullen JM Macdonald J The cytotoxin of *Actinobacillus pleuropneumoniae *(pleurotoxin) is distinct from the haemolysin and is associated with a 120 kDa polypeptide J Gen Microbiol 1991 137 561 568 2033378 10.1099/00221287-137-3-561

[B33] Derijard B Hibi M Wu IH Barrett T Su B Deng T Karin M Davis RJ JNK1: a protein kinase stimulated by UV light and Ha-Ras that binds and phosphorylates the c-Jun activation domain Cell 1994 76 1025 1037 10.1016/0092-8674(94)90380-8 8137421

[B34] Bosshart H Heinzelmann M Targeting bacterial endotoxin: two sides of a coin Ann N Y Acad Sci 2007 1096 1 17 10.1196/annals.1397.064 17405910

[B35] Huether MJ Lin G Smith DM Murtaugh MP Molitor TW Cloning, sequencing and regulation of an mRNA encoding porcine interleukin-1β Gene 1993 129 285 289 10.1016/0378-1119(93)90281-7 8325511

[B36] Baarsch MJ Wannemuehler MJ Molitor TW Murtaugh MP Detection of tumor necrosis factor alpha from porcine alveolar macrophages using an L929 fibroblast bioassay J Immunol Methods 1991 140 15 22 10.1016/0022-1759(91)90121-U 1712031

[B37] Lin G Pearson AE Scamurra RW Zhou Y Baarsch MJ Weiss DJ Murtaugh MP Regulation of interleukin-8 expression in porcine alveolar macrophages by bacterial lipopolysaccharide J Biol Chem 1994 269 77 85 8276881

[B38] Ramjeet M Cox AD Hancock MA Mourez M Labrie J Gottschalk M Jacques M Mutation in the LPS outer core biosynthesis gene, *galU*, affects LPS interaction with the RTX toxins ApxI and ApxII and cytolytic activity of *Actinobacillus pleuropneumoniae *serotype 1 Mol Microbiol 2008 70 221 235 10.1111/j.1365-2958.2008.06409.x 18713318

[B39] Liu H Sidiropoulos P Song G Pagliari LJ Birrer MJ Stein B Anrather J Pope RM TNF-α gene expression in macrophages: regulation by NF-κB is independent of c-Jun or C/EBPβ J Immunol 2000 164 4277 4285 10754326 10.4049/jimmunol.164.8.4277

[B40] Gupta S Campbell D Derijard B Davis RJ Transcription factor ATF2 regulation by the JNK signal transduction pathway Science 1995 267 389 393 10.1126/science.7824938 7824938

[B41] Kyriakis JM Banerjee P Nikolakaki E Dai T Rubie EA Ahmad MF Avruch J Woodgett JR The stress-activated protein kinase subfamily of c-Jun kinases Nature 1994 369 156 160 10.1038/369156a0 8177321

[B42] Bennett BL Sasaki DT Murray BW O'Leary EC Sakata ST Xu W Leisten JC Motiwala A Pierce S Satoh Y Bhagwat SS Manning AM Anderson DW SP600125, an anthrapyrazolone inhibitor of Jun N-terminal kinase Proc Natl Acad Sci USA 2001 98 13681 13686 10.1073/pnas.251194298 11717429 PMC61101

[B43] Thomas SM DeMarco M D'Arcangelo G Halegoua S Brugge JS Ras is essential for nerve growth factor- and phorbol ester-induced tyrosine phosphorylation of MAP kinases Cell 1992 68 1031 1040 10.1016/0092-8674(92)90075-N 1312392

